# Epigenetic Response of *Yarrowia lipolytica* to Stress: Tracking Methylation Level and Search for Methylation Patterns via Whole-Genome Sequencing

**DOI:** 10.3390/microorganisms9091798

**Published:** 2021-08-24

**Authors:** Monika Kubiak-Szymendera, Leszek P. Pryszcz, Wojciech Białas, Ewelina Celińska

**Affiliations:** 1 Department of Biotechnology and Food Microbiology, Poznan University of Life Sciences, 460-637 Poznań, Poland; monika.kubiak1@up.poznan.pl (M.K.-S.); wojciech.bialas@up.poznan.pl (W.B.); 2 Centre for Genomic Regulation (CRG), The Barcelona Institute of Science and Technology, 08003 Barcelona, Spain; lpryszcz@crg.eu

**Keywords:** epigenome, genomics, yeast, next generation sequencing, stress response, methylation level, *Yarrowia lipolytica*

## Abstract

DNA methylation is a common, but not universal, epigenetic modification that plays an important role in multiple cellular processes. While definitely settled for numerous plant, mammalian, and bacterial species, the genome methylation in different fungal species, including widely studied and industrially-relevant yeast species, *Yarrowia lipolytica*, is still a matter of debate. In this paper, we report a differential DNA methylation level in the genome of *Y. lipolytica* subjected to sequential subculturing and to heat stress conditions. To this end, we adopted repeated batch bioreactor cultivations of *Y. lipolytica* subjected to thermal stress in specific time intervals. To analyze the variation in DNA methylation between stressed and control cultures, we (a) quantified the global DNA methylation status using an immuno-assay, and (b) studied DNA methylation patterns through whole-genome sequencing. Primarily, we demonstrated that 5 mC modification can be detected using a commercial immuno-assay, and that the modifications are present in *Y. lipolytica*’s genome at ~0.5% 5 mC frequency. On the other hand, we did not observe any changes in the epigenetic response of *Y. lipolytica* to heat shock (HS) treatment. Interestingly, we identified a general phenomenon of decreased 5 mC level in *Y. lipolytica*’s genome in the stationary phase of growth, when compared to a late-exponential epigenome. While this study provides an insight into the subculturing stress response and adaptation to the stress at epigenetic level by *Y. lipolytica*, it also leaves an open question of inability to detect any genomic DNA methylation level (either in CpG context or context-less) through whole-genome sequencing. The results of ONT sequencing, suggesting that 5 mC modification is either rare or non-existent in *Y. lipolytica* genome, are contradicted with the results of the immunoassay.

## 1. Introduction

DNA methylation is a common, but not universal, epigenetic modification that plays an important role in multiple cellular processes, including genome structure rearrangements [1,2,3], development [2,4,5], or the response to environmental factors [6,7]. DNA methylation relies on covalent addition of methyl groups to cytosine (5 mC), and less frequently—to adenosine (6 mA) or to uracil (hydroxyl-mU; the latter is specific for dinoflagellates) [8]. In eukaryotes, the modification typically occurs at the 5-position of cytosine residues (5 mC) in CpG dinucleotide patterns. The occurrence of 5 mC is widespread in the majority of prokaryotic (bacterial, archaeal) [9] and eukaryotic genomes, including plants and animals [10,11,12]. However, genome methylation in different fungal species is still a matter of debate. The occurrence of this epigenetic modification has been confirmed by different technical approaches in several yeast species, including *Komagataella phaffii* [13], *Candida albicans* [1], and *Saccharomyces cerevisiae* [14,15]. On the other hand, the vast majority of reports deny the presence of genomic DNA methylation in *S. cerevisiae* [8,16] and *K. phaffii* [13], and many other yeast species.

The chemical modification of DNA and the proteins involved in genome maintenance (in particular histones) impact chromatin structure. Chromatin state (active or repressive) plays a major role in the regulation of gene expression due to controlling the access of the transcriptional machinery to DNA [17]. Initially, it was presumed that only transposable elements (TEs) and repetitive sequences are subjected to methylation events, which was considered a cellular strategy to assure genome stability. However, recent studies have shown that promoters and transcribed regions can be methylated as well [2,5,9,18]. Hypermethylation, located at a promoter region around the transcription start site, leads to gene repression [19], hence is generally regarded as a silencing epigenetic mark [20]. Notably, based on research into heterologously overexpressed DNA methylases in a system natively lacking genomic DNA methylation, it was inferred that DNA methylation can significantly affect transcription even in the absence of co-evolved cellular machinery interacting with DNA methylation [13].

The epigenome (the total of genomic DNA modifications) is plastic and flexible, as DNA methylation is a reversible biological signal. The density and pattern of DNA methylation are tightly connected to changes in gene expression, massively occurring upon developmental changes or through other specific whole-cell transitions, such as, for example, morphogenesis [2,4,14]. In addition, epigenetic alterations can be induced by environmental factors, in particular, under stress conditions (thermal stress, oxidative stress, osmotic stress, etc.) [7,21,22,23]. Microbial cells induce stress responses to adverse conditions resulting in the activation of a wide range of proteins involved in stress response, cell rescue, and metabolism [24,25]. Demethylation of stress-induced genes in response to cold has been observed in the psychrophilic yeast species *Naganishia antarctica* [22]. In this sense, the DNA methylation pattern provides a biological link between the environmental exposures of individuals and their phenotype. It is still unclear whether such epigenetic patterns can be inherited by nascent generations. Several studies reported epigenetic inheritance in mammals, plants, and fungi [23,26,27]. Recent results indicate that most of these stress-induced modifications reverse to their original methylation status after the stress cessation and/or across several generations [7,28]. In contrast, other reports support the inheritance of the epigenetic traits as a “stress memory” [11,29]. Apart from DNA methylation, other epigenetic modifications including histone modification contribute to the hereditary transmission of chromatin states, and the gene activation or silencing [23,30]. The epigenetic inheritance of telomeric and centromeric silencing in *S. cerevisiae* and *Schizosaccharomyces pombe* has been studied and confirmed [31]. Despite the advances in the knowledge on environmentally induced changes in DNA methylation, it remains unclear what is the extent of their transgenerational inheritance. The fact that DNA methylation is inheritable and has an impact on phenotype suggests that the mechanism could play a role in natural selection and adaptation.

*Yarrowia lipolytica* is one of the most studied non-conventional yeast species with a promising biotechnological potential [32,33]. Like other yeast species, *Y. lipolytica* cells undergo massive changes in metabolism and morphology in response to environmental conditions [34,35,36]. Recent studies on genomic DNA methylation in *Y. lipolytica* exploiting the GC-MS technique proved the occurrence of 5 mC modification in this species [15]. Earlier, dimorphic transition in *Y. lipolytica* was shown to be correlated with differential DNA methylation patterns, as demonstrated by methylation-sensitive amplified polymorphism (MSAP) [10]. In the former study, 5 mC levels were shown to vary considerably at different growth stages [15]. While changes in the total proteome in *Y. lipolytica* under osmotic stress have been analyzed [24], no studies on epigenetic changes in the genome under stress responses have been published to date.

In this paper, we report on differential DNA methylation levels in the genome of *Y. lipolytica* subjected to sequential subculturing and to heat stress (HS) conditions. Additionally, we address the question of whether the stress-induced DNA methylation level/pattern is inherited by nascent generations. To this end, we adopted repeated batch bioreactor cultivations of *Y. lipolytica* repeatedly subjected to thermal stress. To analyze the variation in DNA methylation between stressed and control cultures, we (a) quantified the global DNA methylation status using an ELISA-based assay, and (b) studied DNA methylation patterns through Oxford Nanopore Technology (ONT) whole-genome sequencing. Ultimately, this study provides an insight into the stress responses and adaptation of the yeast *Y. lipolytica* to the stress factors at the epigenetic level.

## 2. Materials and Methods

### 2.1. Strain

A French line, wild-type, type *Y. lipolytica* strain W29 (CLIB89; ATCC20460) was used in this study. The complete genome sequence of this strain was obtained from JGI MycoCosm [37,38] (https://mycocosm.jgi.doe.gov/YarliW29_1/, accessed on 25 March 2019).

### 2.2. Preculture Development

Precultures cultures were developed from glycerol stocks of *Y. lipolytica* W29 stored at −80 °C, by spreading the biomass on YPD medium (g L^−1^): yeast extract (BTL, Lodz, Poland), 10; bactopeptone (BTL, Lodz, Poland), 20; glucose (POCh; Avantor Performance Materials Poland, Gliwice, Poland), 20; agar (BIOCORP, Paris, France), 20. Afterwards, a loop-full of the biomass was transferred into a 1-L Erlenmeyer flask containing 100 mL of YPG_20_ medium (g L^−1^): yeast extract, 10; bactopeptone, 20; glycerol (POCh; Avantor Performance Materials Poland, Gliwice, Poland), 20. Flask cultivations were conducted in an orbital shaker incubator (BIOSAN; Riga, Latvia) at 250 rpm at 30 °C for 23 h.

### 2.3. Bioreactor Culture Conditions

All of the cultures were conducted in BIOSTAT^®^ B plus stirred-tank bioreactors (Sartorius; Germany), with a total volume of 5 L and 1 L working volume in YPG_120_ culture medium (composed as follows (g L^−1^): yeast extract, 10; bactopeptone, 20; glycerol, 120). 0.9 L of that medium was inoculated with 100 mL of the 23 h *Y. lipolytica* W29 preculture. pH, temperature, stirring, and aeration were maintained automatically at 5.5 (with 10% vv^−1^ H_2_SO_4_ and 40% NaOH vv^−1^ as acidic and basic regulators), 28 °C, 400 rpm, and 2 vv^−1^m^−1^, respectively. AntiFoam 204 (Merck-Sigma-Aldrich; St. Louis, MO, USA) was used as a defoaming agent. The bioreactor cultures were carried out in two different modes, batch and repeated batch, each in a variant subjected and non-subjected to thermal stress. Each variant of culture was conducted in three independent runs.

### 2.4. Batch and Repeated Batch Cultivations

Batch (B) cultures were conducted for 24 h in 1 L working volume. Samples were collected at 20 (before subjection to thermal stress), 22 and 24 h of culture (1 h and 3 h after the end of heat-shock; HS), and immediately centrifuged (5400× *g*, 3 min; Eppendorf MiniSpin, Hamburg, Germany).

Repeated batch (RB) cultures were conducted in three 26-h cycles (78 h in total) in 1 L working volume. Each of the cultivations was inoculated at 10% vv^−1^. The standard preculture (developed as described above) was used for inoculation of the first cycle, whereas 100 mL of a previous culture liquid constituted a preculture for the next cycle. For the first 26 h, the cultivation was carried out in a batch mode, then, 90% of the culture was withdrawn, and the same volume of the fresh YPG_120_ medium was added. Samples were collected at 20 (before subjection to thermal stress), 22, and 26 h of culture (1 h and 5 h after the end of HS), and immediately centrifuged (9000 rpm, 3 min; Eppendorf MiniSpin, Hamburg, Germany).

### 2.5. Heat Stress Conditions

Thermal stress treatment was executed by transferring the culture (using a peristaltic pump; Watson-Marlow 323; Watson Marlow, Falmouth, UK) from the bioreactor to a sterile 2-L bottle placed in a 42 °C water bath with stirring and oxygen provision. The stress was applied at 20 h of culturing and maintained for 1 h, which was pre-determined by analyzing biomass growth and glycerol consumption by the strain in YPG_120_ medium. At that point, the culture was in the late exponential growth phase, which has been previously shown as being optimal for thermal stress execution [39].

### 2.6. Isolation of Genomic DNA, RNAse Treatment and Determination of Global DNA Methylation Level

Genomic DNA of *Y. lipolytica* W29 strain was isolated using the YeaStar Genomic DNA Kit (Zymo Research, Irvine, CA, USA) according to the manufacturer’s protocol. The optional step with chloroform addition was included in the procedure to increase DNA recovery. To remove possible RNA contamination from genomic DNA isolates, the preparations were treated with RNAse A (ThermoFisher Scientific, Waltham, MA, USA) for 1 h at 37 °C, according to the manufacturer’s recommendation. The resultant DNA preparations, devoid of RNA, were precipitated with ethanol and purified (Clean-Up; A&A Biotechnology, Gdansk, Poland). The quantity and quality of the genomic DNA were verified through agarose-gel electrophoresis and spectrophotometric measurement (NanoDrop; Thermo Fisher Scientific, Waltham, MA, USA).

The genome-wide DNA methylation level (5 mC) was determined using the Methylated DNA Quantification (Colorimetric; Abcam, Cambridge, UK, ab117128) kit, which is an ELISA-type assay for detecting 5-methylcytosine. All reactions were set up according to the protocol recommended by the supplier, including titration of positive control (DNA methylated at a specified percentage) and negative control (DNA devoid of any methylation). The amount of DNA added to the reaction mixture was each time standardized to 100 ng. Prior to the 5 mC (%) calculation, the amount of 5 mC (ng) was quantified using the slope (OD450 nm/ng) of the standard curve. The global DNA methylation was calculated using the following formula:

(1) $$5\;\mathrm{mC}\%\;=\;\frac{5\;\mathrm{mC}\;\mathrm{amount}\;(\mathrm{ng})}{\mathrm{amount}\;\mathrm{of}\;\mathrm{input}\;\mathrm{sample}\;\mathrm{DNA}\;(\mathrm{ng})}$$

All results were normalized to the readout for negative control (DNA devoid of any methylation) provided by the kit’s manufacturer, by subtraction of the control sample value from the readout for the unknown samples. The results were expressed as the mean values ± SD of the three independent biological replicates, all analyzed in technical duplicate.

### 2.7. Whole Genome ONT Sequencing and Search for DNA Methylation Patterns

High-Throughput Nanopore Sequencing was used for the mapping of DNA methylation patterns across the whole genome using a Ligation Kit (Oxford Nanopore Technologies; ONT, Oxford, UK). Raw reads were basecalled, quality filtered, and de-multiplexed using guppy basecaller v3.6.1. Raw sequencing data are available in the NCBI SRA under PRJEB44499 accession. Methylation events were analyzed using modPhred (https://github.com/novoalab/modPhred, accessed on 8 July 2021) applying two DNA modification-aware basecalling models: (1) dam-dcm (distributed with guppy v3.6.1) that is able to report m5C in CpG and CCWGG contexts, and N6mA in GATC contexts, and (2) res_dna_r941_min_modbases-all-context_v001.cfg (available via https://github.com/nanoporetech/rerio/, accessed on 28 July 2021) that is able to report m5C and N6mA in a context-independent manner.

### 2.8. High-Performance Liquid Chromatography

The concentration of small-molecular weight metabolites (erythritol, mannitol, citric acid, alpha-ketoglutaric acid) and the carbon source (glycerol) in the culturing medium was measured by HPLC. Collected samples were centrifuged and the supernatants were filtered through 0.45 μm membrane syringe filters (Millipore; Merck-Millipore, Burlington, MA, USA). The chromatograph Elite LaChrom, VWR-Hitachi equipped with two detectors (RI L-2490 and UV L-2400) was used. The Rezex ROA 300 × 7.8 mm column (Phenomenex) was calibrated using standard solutions of the chemicals (Sigma-Aldrich Co., St. Louis, MO, USA). The analyses were conducted at 40 °C, under isocratic conditions, with a flow rate of 0.6 mL min^−1^ and 10 mM H2SO4 as the mobile phase. The compounds in the samples were quantitatively analyzed in reference to the standard solutions (peak area) using EZChrom Elite (Agilent) software 3.2.0.

### 2.9. Statistical Analysis

Obtained results were expressed as the mean ± standard deviation (±SD) of the replicates (three biological repetitions, each analyzed in technical triplicate). The statistical importance of the differences between compared sets of data was analyzed using one and multi-way analysis of variance (ANOVA and Tukey’s multiple comparison tests (Statistica 13 PL; Statsoft Inc., Krakow, Poland). For repeated-batch cultures, the significance of the main effects of: (1) heat shock, (2) time after HS, and (3) number of cycles, was assessed (the interactions were not significant). The levels of significance were set at *p *< 0.005 or *p *< 0.01 (indicated). Details of statistical analysis are given as Supplementary File 3: Table S1. Graphical presentation of the obtained data was carried out using Microsoft Excel 2013 software.

## 3. Results

### 3.1. Global Level of Genomic DNA Methylation in Y. lipolytica Batch Cultivations

In the present study, we used an ELISA-based immunoassay for preliminary estimation of the global genomic DNA methylation level in *Y. lipolytica*. The assay is specific towards 5 mC detection by exploitation of a primary antibody raised against 5 mC, and a labeled secondary antibody, which enables colorimetric detection. According to the manufacturer’s information, the kit possesses a detection limit of ≥0.2 ng of methylated DNA per reaction. The immunoassay kit (ab117128) used in this study was previously used to quantify global DNA methylation in mammalian cell lines [40], adult *Drosophila melanogaster* (~0.4% of 5 mC) [41], and *Paramecium tetraurelia* (unicellular eukaryote) at different developmental stages (~0.2–2.2% of 5 mC) [42]. However, the latter organism results obtained using the antibody-based method were contradicted by DNA mass spectrometry and bisulfite sequencing analyses.

**Figure 1:**
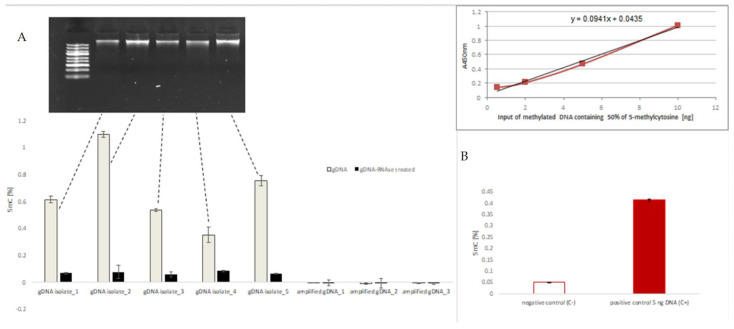
Frequency of 5 mC [%] in (**A**) nucleic acid isolates from *Y. lipolytica* W29 strain and in PCR-amplified DNA template prior (grey) and after (black) RNAse A treatment; upper panel shows electrophoretic separation of the genomic DNA (gDNA) preparation in 1% agarose gel (M: Gene Ruler Express; Thermo Fisher Scientific); (**B**) positive and negative control samples provided by the immunoassay manufacturer; upper panel–standard curve for the assay showing its linearity and detection ranges; Negative control was an unmethylated polynucleotide containing 50% of cytosine. Important: the value of the 5 mC (A450 nm) read for the negative control sample (C-) was subtracted from all the analyzed samples in (**A**). Error bars indicate the ±SD from biological triplicate, each analyzed in technical triplicate.

In the present study, we first set a series of batch cultivations (the substrate and metabolites concentrations are given in Supplementary File 1: Figure S1A,B) to evaluate the usefulness of the assay for *Y. lipolytica*’s genomic DNA preparations isolated from HS-treated and control variants, since, as discussed by McRae et al. [26], the immunoassays are useful solely for evaluation of relatively large changes in the DNA methylation level (of 1.5–2-fold). Primarily, our initial 5 mC estimates (~3%; not shown) were on average 10-fold higher than in a recent study reporting genomic DNA methylation in *Y. lipolytica* revealed using GC-MS (0.3%) [15]. On the other hand, the present results fall within the scope of 5 mC frequency levels determined for different fungal species revealed by reverse phase-HPLC [43] or MSAP [1]. Since the immunoassays are also known to give false-positive results with methylated RNA, we set an experiment verifying the potential contamination of the genomic DNA preparations with methylated RNA. The experiment covered randomly selected genomic DNA preparations from different time-points of the HS/control cultures and amplified genomic DNA (devoid of methylation) prior, and after treatment with RNAse A, as well as positive and negative controls from the kit’s provider (Figure 1A,B). Strikingly, it turned out that indeed the positive signal from 5 mC immunoassay in the genomic DNA preparations came mainly from methylated RNA contaminations. RNA was not seen in the electrophoretic images of the isolated nucleic acids (Figure 1A), indicating that its concentration in the preparation was low, but obviously, that was the main, methylated fraction of the isolated nucleic acids. Those preliminary studies showed that even if extracted and purified with a DNA-specific kit, the genomic DNA preparations to be analyzed for 5 mC level with the immunoassay must be decontaminated from residual RNA to eliminate false-positive results (amounting up to 10-fold). Taking this into account, we conclude that the immunoassay-detected 5 mC level in *Y. lipolytica* genomic DNA was on average ~0.05%

### 3.2. Global Level of Genomic DNA Methylation in Y. lipolytica Repeated-Batch Cultivations

**Figure 2:**
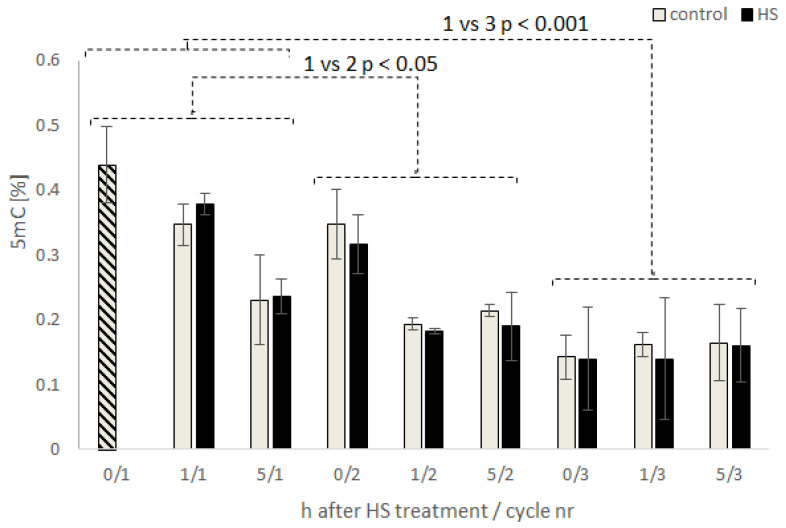
Level of global DNA methylation (5 mC) in *Y. lipolytica* W29 strain cultivated in repeated-batch cultures, under HS (grey) and control (black) conditions. Sample names are encoded: hours after HS/number of cycles. Error bars indicate the ±SD from biological triplicate, each analyzed in technical triplicate.

The detection of 5 mC in *Y. lipolytica* genomic DNA and the feasibility of its assaying with the immunoassay were the prerequisites for the following experiments, addressing the questions of this species’ epigenetic response to HS treatment, and transgenerational inheritance of the awakening response. To address those issues, we cultured *Y. lipolytica* in repeated-batch (RB) mode with intermittent exposition to HS conditions, administered at a predetermined intensity and period. Time, and conditions of the HS administration (20 h; late exponential growth phase; 1 h at 42 °C) were established based on a previous detailed study on *Y. lipolytica* adaptation to heat treatment [39] and our own preliminary experimentation. Altogether, the RB cultures were continued throughout three consecutive batches (cycles). Each subsequent batch was inoculated at 10% with the cell population from the previous batch (either stressed–HS, or not–control). Samples withdrawn from the cultures were analyzed for global methylation level of genomic DNA after RNAse treatment. In each cycle, 5 mC level was analyzed directly prior to the HS (0/…; at 20 h of culturing), as well as 1 h (22 h) and 5 h (26 h) after the HS treatment (1/… and 5/…, or at the corresponding time-points in the control). The results of this analysis are shown in Figure 2.

Primarily, we did not observe any specific, physiological reaction in *Y. lipolytica* cells to the adopted HS treatment in terms of 5 mC level (Figure 2; Supplementary File 3; *p* < 0.05), glycerol utilization kinetics, nor synthesis of the main metabolites (Supplementary File 2: Figure S2A,B). The only exception was a significant difference in the concentration of erythritol, which was 1.66-fold lower in the HS-treated variant in the first RB cycle when compared to the control. While its concentrations were very low (3.8–6.3 g/L), it is known that modulation in the polyols synthesis is a marker of stress. Erythritol synthesis is stimulated by enhanced medium osmolarity [24], and recently we observed significant modulation in mannitol synthesis upon burdening *Y. lipolytica* cells with high-level overproduction of secretory proteins [44].

In contrast, we observed that the 5 mC level changed with the time of the culture duration within the first two cycles of RB (/1 and /2 by 1.6–2-fold; details of statistical significance analysis are given in Supplementary File 3: Table A,B in Table S1). Corresponding observations were reported for *Y. lipolytica* grown in the exponential and stationary phase of growth, where 5 mC was determined by GC-MS [15]. In that study, over a 2-fold decrease in 5 mC level was recorded for the cells entering the stationary phase of growth, when compared to the cells in the exponential growth phase; which well corresponds with the values recorded in the present research. Based on our current results and those previous studies, it could be speculated that upon entering the stationary phase of growth, *Y. lipolytica* cells reduce the level of genomic DNA methylation. Notably, this observation was valid irrespective of the HS administration, which implies that either HS does not impact the genomic DNA methylation level in *Y. lipolytica*, or that the impact of HS was superseded by the effects triggered by the growth phase shift; or some other, undetermined factors.

Notably, our current data indicate that with every repetition of the batch cultivation (either stressed or not), the magnitude of the drop in 5 mC level between 20 h and 26 h of culturing in a given cycle (prior and post HS) and the absolute quantities of 5 mC were both significantly reduced (at *p* < 0.01 and *p* < 0.005 for comparisons cycle 1 vs. 2, and cycle 1 vs. 3, respectively; Supplementary File 3: Table A,B in Table S1; Figure 2). Surprisingly, in the late exponential growth phase (20 h; point 0/) of the 3rd cycle, the level of genomic DNA methylation was drastically reduced when compared to the two former cycles (2.6-fold in HS, 2.71-fold in the control culture). Moreover, in that cycle, no changes in 5 mC frequency depending on the batch duration were observed (~0.15 % of 5 mC throughout the batch), indicating that the general trend for reduction of genomic 5 mC with the time of cycle duration frequency was lost. A lower frequency of 5 mC at the late exponential growth phase of the 3rd cycle suggests that sequential subculturing imposed an environmental pressure that favors genomes with low levels of methylation. It could be equally well induced by accumulating by-products of metabolism, transferred with an inoculum to the next batch (i), or passed within the genetic material (ii). The question is whether it was a real-time reaction to the environmental conditions (?) (i), or it was inherited from the mother cells as (epi)genomic rearrangements (?) (ii). We approached that problem by using ONT whole-genome sequencing (Section 3.3).

**Figure 3:**
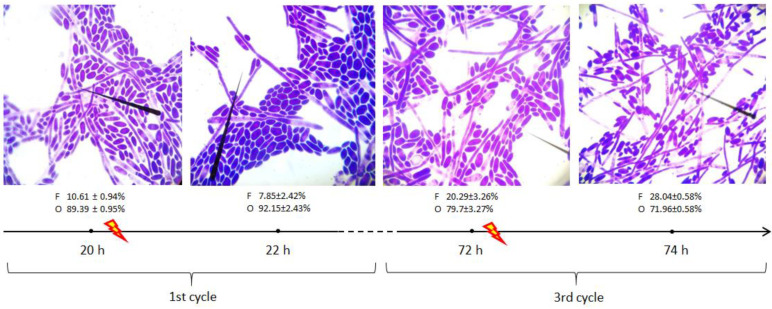
Representative images from light microscopy observations of *Y. lipolytica* cells in repeated-batch cultures at different stages. Magnification 1000× (PrimoStar, Carl Zeiss light microscope). Mean percentage of filamentous (F) and ovoid forms (O) ± SD from five microscopic preparations.

Except for tracking changes at the genome and epigenome levels, stability and condition of the culture can be inspected by the visual appearance of the cells throughout subculturing, which is the most common in practice. *Y. lipolytica*, as a dimorphic species, can grow in ovoid or filamentous morphotype, with a full scope of intermediate morphologies [45,46]. Dimorphic transition in this yeast species is tightly coupled with genome-wide alterations [10,47]. It is known that filamentation by *Y. lipolytica* cells is a marker of the ongoing stress response [45,48,49,50], but in order to occur, requires specific conditions [51]. To get an insight into this aspect of the culture state, we analyzed the cells’ morphology by light microscopy and calculated the relative number of filamentous and ovoid morphotypes (Figure 3). The obtained results strongly suggest that it was the repeated subculturing and not the HS treatment that significantly contributed to dimorphic transition. The number of filaments in cycle 1 prior and after the HS is not significantly different (10.68 ± 0.94 vs. 7.85 ± 2.42%), on the other hand, the share of filamentous forms was over 2-fold higher at the beginning of cycle 3 (20.29 ± 3.26%). The HS treatment in the 3rd cycle of RB contributed to a further increase in filamentation. Such an observation supports our results on changed (decreased) global level of 5 mC frequency, detected with immunoassay (Figure 2).

Based on these results we inferred that *Y. lipolytica*’s genome is subjected to DNA methylation, *Y. lipolytica* responds with its genomic DNA methylation status and morphology to the repeated subculturing but not to the HS treatment, and that the adopted culturing conditions are adequate for awaking epigenetic responses in its genome.

### 3.3. Whole Genome ONT Sequencing in the Search for Genomic DNA Methylation Pattern in Y. lipolytica Repeated-Batch Cultures

In order to obtain a deeper insight into specificities of those epigenetic modifications, we used the ONT whole-genome sequencing for the samples withdrawn from the control RB cultures and the HS-treated RB cultivations, during the 1st and the 3rd batch of RB cultivations, prior to, 1 h and 5 h after the HS treatment (or the corresponding time-point of the control cultures). Even though we did not see any significant changes in the global level of genomic DNA methylation due to HS treatment, we decided to comparatively analyze the epigenetic patterns, as we presumed that even if the global methylation level was not significantly changed, the pattern of methylation could be modified (which cannot be assayed using the immunoassay). A PCR-amplified genomic DNA, devoid of methylation, was used as a background control.

**Figure 4:**
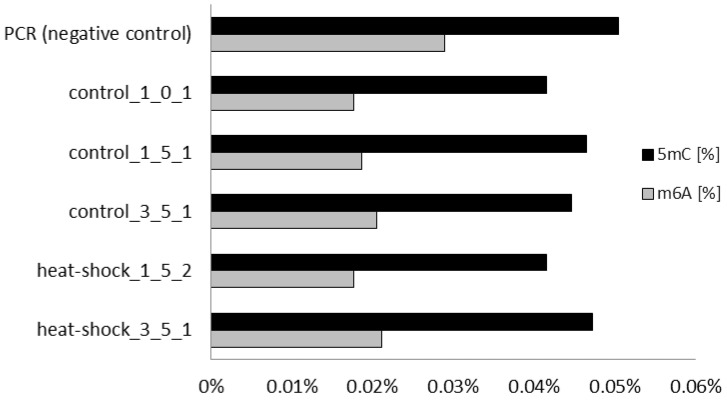
The level of global DNA methylation (5 mC and N6mA) using dam/dcm and CpG model (ONT Guppy basecalling software version 3.6.1). Sample names are encoded: hours after HS/number of cycles.

The first set of bioinformatics analyses was oriented towards the search for transitions of 5 mC/C that are localized within the CpG dinucleotide patterns. Additionally, models trained in the detection of bacterial dam/dcm methylation patterns were adopted as well. The relative level of 5 mC and N6 mA within the CpG context of genomic DNA of *Y. lipolytica* cultured in RB cultivations, subjected or not to HS, is shown in Figure 4.

**Figure 5:**
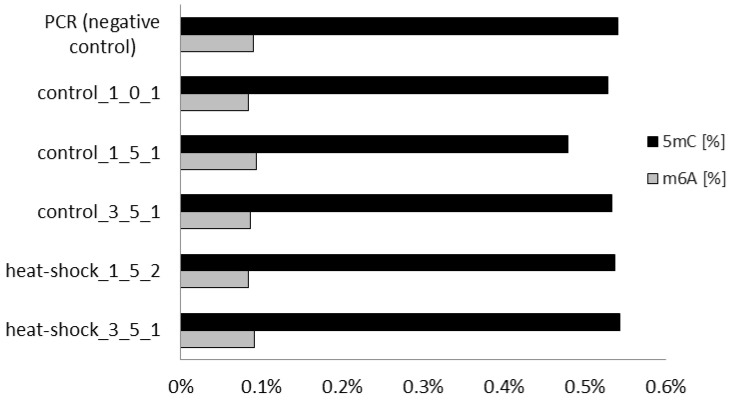
Level of global DNA methylation (5-methylcytosine and N6-methyladosine content) in all contexts (ONT Guppy basecalling software version 3.6.1). Sample names are encoded: hours after HS/number of cycles.

Unexpectedly, the adopted search strategy did not yield any response (detect any 5 mC or N6mA) within the analyzed genomic DNA preparations that would be higher than the background control level (PCR-amplified negative control). Neither the models that were trained to detect methylation in bacterial (dam/dcm) nor eukaryotic (CpG) contexts could not detect any methylation in *Y. lipolytica* genomic DNA (Figure 4). It was very surprising, especially that previous studies clearly indicated that patterns which are methylated in *Y. lipolytica* are typical CpCpGpG, that could be revealed by the MSAP technique [10]. The CpG context is considered a universal methylation site within eukaryotic genomes. On the other hand, a recent study conducted in *K. phaffii* heterologously overexpressing human DNMTs showed that different DNMTs have distinct patterns of sequence preference and aversion [13]. That phenomenon was associated with the sequences flanking centrally located CpG, and their impact was stronger or weaker depending on the DNMT isoform. In addition, it has been previously estimated that approximately 20% of individual differences in DNA methylation in the population are caused by DNA sequence variation that is not located within CpG sites [26]. Therefore, we decided to undertake a different approach and to search for the DNA methylations in a context-independent manner. The results of the context-independent search for 5 mC and N6mA are shown in Figure 5.

To our surprise, no differences could be detected in the C and A methylation levels between individual DNA samples collected from HS-treated and control RB cultures, also when searched without context restrictions. However, this time, the resultant percentages of 5 mC were at the levels detected using the immunoassay (~0.15–0.5 [%]). Nevertheless, the detected level of 5 mC in the DNA isolates was comparable to the one found in PCR-amplified control (devoid of 5 mC per se), which suggests that the detected methylation was a false-negative result.

## 4. Discussions

According to the current state of the art, DNA methylation in fungi, and specifically–in yeast, is questionable. Depending on the assaying technique and its detection limit, the genomic cytosine modification is either detected or not. Until the work by Hattman et al. [14], methylation of genomic DNA in yeast had not been definitely settled. Using radioisotope labeling of DNA, followed by acid hydrolysis and thin-layer chromatography (TLC), 5 mC was identified in genomic DNA of *S. cerevisiae*; but the estimation of 5 mC amounts was highly variable (0.3–1.0 5 mC per 100 C residues) [14]. The two following studies by Proffitt et al. [8] and Antequera et al. [16] exploited the most popular techniques for 5 mC detection in genomic DNA, namely MSAP, followed by either HPLC or TLC of radiolabeled nucleotides, respectively. None of the approaches applied, allowed detection of genomic cytosine methylation in i.a. *S. cerevisiae*, *Sch. pombe*, or, closely related to *Y. lipolytica*, *Aspergillus nidulans*. Reverse-phase HPLC was also used to quantify 5 mC in several fungi species [43]. While the technique was applied to detect and measure methylated cytosines in five of the species (at 1.3–4.3%), no 5 mC was detected in *S. cerevisiae* [43]. Using the MSAP technique, Mishra et al. [1], not only detected 5 mC in *Candida albicans*, but also quantified it (0.5% 5 mC), and revealed by sequencing that the modification is largely localized to structural genes. However, as discussed there, the detected level of methylated cytosines in *C. albicans* was relatively low, when compared to the other fungal representatives (2.3–4.3% of 5 mC). Recently, tandem mass spectrometry coupled with liquid chromatography (LC-MS/MS) was exploited to quantify 5 mC in an array of *Saccharomyces* spp., including several strains of *cerevisiae*, *boulardii*, and *paradoxus* species, but also *Sch. pombe* and *K. phaffii* [44]. While the technique was found adequate (5 mC were identified in reference DNAs of plant, bacteria, and vertebrate origin) no genomic 5 mC was identified in any of the yeast species. On the other hand, MS preceded by gas chromatography (GC-MS) enabled identification and quantification of 5 mC modifications in all nineteen yeast species, including *Candida* spp., *Debaryomyces* spp., *Komagataella* spp., *Saccharomyces* spp., and also *Y. lipolytica* [15]. Notably, the detected level of cytosine methylation was the highest in *Y. lipolytica*, reaching up to 0.36%. Nevertheless, according to those findings obtained by GC-MS, even the highest 5 mC level in yeast was still much below the levels detected in mammals, reaching 8%. Similarly, a very low level of genomic DNA methylation was found in the most recent paper on 5 mC occurrence in yeast, as revealed by whole-genome bisulfite sequencing (WGBS) [13].

The observed high discrepancies in the 5 mC detection and quantitation in yeast genomic DNA cannot be attributed solely to the detection limits of the specific methods. The 5 mC content determined by Hattman et al. [14] much exceeds the sensitivity of LC-MS/MS (0.002%) [52], MSAP, or GC-MS (6.4 fmol). The researchers thus speculated that in some fungal species, genomic DNA methylation is probably dispensable or successfully replaced by the other regulation strategies; and even closely related species are subjected to significant variation in 5 mC frequency/occurrence [16]. Likewise, in a recent study exploiting high-throughput transcriptomics (RNAseq) and (epi)genomics (WGBS), it has been indicated that *K. phaffii* lacks endogenous DNA specific methyltransferases (DNMTs) and genomic DNA methylation, which corresponds with the conclusions by Capuano et al. [52], but contradicts those reached by Tang et al. [15]. Taking advantage of the 5 mC-less genomic background, the researchers overexpressed several different human DNMTs and studied the action of heterologous methylation enzymes in a methylation-less system [13]. Interestingly, it was revealed that even if the cloned enzymes were mammalian (high 5 mC) the global methylation rates were much lower in the engineered yeast than those found natively in mammals; clearly demonstrating that the level of methylation is not related to the “activity” of DNMTs but some other regulatory mechanism. The accompanying transcriptomics revealed that the host yeast cells evoked a transcriptional response that countered the heterologous DNA methylation stress by limiting the abundance of SAM (methyl group donor). It was a fundamental finding—that the cells considered to be devoid of intrinsic DNA methylation are still able to detect DNA methylation stress and mitigate this stress by modulating SAM turnover; moreover, that SAM involvement is conserved across a diverse group of eukaryotes, including those lacking endogenous DNMTs. On the other hand, maintaining a DNA methylation-sensing and -regulating system in a methylation-less biological system seems a waste of energy and building blocks. Hence, it is plausible that some other, still unknown DNA methylation patterns and systems operate in at least some of the yeast species, or that RNA/protein-specific methylation machinery could be hijacked for sensing and regulation of the heterologous DNMTs action. Lessons from *Sch. pombe* show that the cell devoid of classic genomic DNA methylation can bear a (DN)MT active towards specific tRNA and not towards DNA, but the methylation-related system operates in those cells [53].

In the present study we attempted to: (i) verify the occurrence of DNA methylation in genomic DNA of *Y. lipolytica* by using two different techniques—the immunoassay and ONT WGS, which were not previously used in combination with this yeast species; (ii) investigate the epigenetic response of the yeast to environmental stress and repeated subculturing, with the aim to approach the issue of transgenerational inheritance of the developed methylation pattern. Primarily, our results from the immunoassay showed that, indeed, *Y. lipolytica*’s genome is subjected to 5 mC epigenetic modification and that the recorded frequency of 5 mC (~0.15–0.5%) is consistent with the results obtained by GC-MS analysis [15]. We also demonstrated that the immunoassay is specifically prone to render false-positive results due to unexpected contamination with methylated RNA, which must be removed prior to the analysis (Figure 1). RNA methylation is a biological process known to actively respond to various perturbations. Recently, we observed its significant upregulation in response to high-level synthesis and secretion of recombinant secretory proteins in *Y. lipolytica* [54]. Hence, its presence in the samples analyzed for genomic DNA methylation triggers significant, uncontrolled variation to the results.

To study the epigenetic response of *Y. lipolytica* to stress factor treatment and its potential inheritance, we adopted RB cultivations with intermittent exposure to pre-optimized HS. The obtained results clearly indicated that neither HS treatment nor the time after treatment (within a single cycle) has a significant impact on the global 5 mC level in *Y. lipolytica* genome (Figure 2; Supplementary File 3: Table A,B in Table S1). On the other hand, repeated subculturing (consecutive batches inoculated with the same yeast cells population) indeed strongly modulates the global methylation level of genomic DNA. RB cultivations offer a multitude of technological advantages (lower investment cost, shortened batch time, etc.), but also bring about very specific limitations (aging of the culture, genetic instability, inheritance of accumulated mutations, genetic drift). It is well established that excessive subculturing (>5 times, according to ATCC; www.atcc.org) of a microbial strain leads to a loss of its original qualities due to accumulation of point mutations, short (<100 bp) insertion/deletions, but also larger genomic rearrangements, proved by for example PFGE [55]. Subculturing of several yeast strains over many generations, followed by whole-genome DNA sequencing, has allowed a more global analysis of the genomic alterations and their quantitative characterization [56]. Repeated subculturing of entomopathogenic fungus leads to attenuation of its desired traits [57]. The authors suggested that the background behind that transition was most probably the inheritance of altered characteristics involving DNA methylation, transposon activity, and dsRNA viruses. Those results well align with our current observations, indicating that repeated subculturing indeed triggers a significant epigenetic response in the *Y. lipolytica* genome. The immunoassay data supporting that statement are additionally backed up by microscopic observations (Figure 3). It was previously reported by Mishra [1] that the genes involved in dimorphic transition were particularly enriched in 5 mC modification. It can be speculated that upon repeated subculturing, the genes responsible for dimorphic transition, such as the master regulators FLO11 and MHY1 and their downstream targets, were demethylated, which contributed to enhanced filamentation.

Previous research into the relationship between genomic DNA methylation and fungi cells’ response to environmental stress conditions report on highly species-specific outcomes. It was, for example, shown using MSAP that changes in the available carbon source significantly changed MSAP banding profiles in Metschnikowia reukaufii, indicating changes in the epigenome [21]. Two *Naganishia* species: *N. albida* and *N. antarctica*, responded to cold stress with either a nonsignificant increase or nonsignificant decrease in genome methylation level [22]. Interestingly, after cessation of the stress conditions, the changed methylation profile was reversed to the control state in *N. antarctica*; and increased up to a significant level in the second species. However, more insightful studies into specific cold stress-related loci showed that after recovery of the control conditions, the loci that were demethylated in response to cold stress were remethylated again, after the stress factor cessation, which directly indicates their involvement in the stress response. In terms of our results, one can state that *Y. lipolytica* showed chimeric behavior compared to the two *Naganishia* spp. (Figure 2). Depending on the RB cycle, its genomic DNA methylation status was either recovered to the original state (cycle 1/2; such as *N. antarctica*) or continued epigenetic rearrangements (cycle 2/3; like N. albida).

Definitely, the most unexpected result of the present study was the inability to detect 5 mC (and 6 mA), at the level above the PCR-amplified negative control, in genomic DNA of *Y. lipolytica*, irrespective of the treatment/culturing time, or adopted search mode (context-sensitive or context-less). In contrast to the immunoassay, ONT sequencing could not be hampered by RNA contamination, simply considering the mechanism of the ONT sequencing reaction (PCR, the addition of a sequencing adapter and motor protein, mapping both strands of the genome, etc. all require DNA template). Thus, the unexpectedly equal result for the negative control and all the analyzed samples (Figure 4 and Figure 5) do not result from false-positive signals from methylated RNA. In addition, the adopted ModPhred model was previously found useful and highly reliable in the detection of 5 mC and N6mA modification in microbial genomes when fed with reads basedcalled by a modification-aware basecaller [58]. Current guppy basecalling models (versions 3.2.1 and later) can detect m5C in CCWGG and CpG contexts, and m6A in GATC contexts, which was considered sufficient for analysis of *Y. lipolytica* genome. At present, the contradictory results of the immunoassay and ONT whole-genome sequencing are unclear, especially that the same type of sample (PCR-amplified *Y. lipolytica* genomic DNA negative control) was used to normalize both techniques (see bars for “amplified gDNA” in Figure 1). The same sample of PCR-amplified genomic DNA gave no detectable 5 mC in the immunoassay (as could be expected), and 0.05 or 0.5% 5 mC in the ONT sequencing (depending on the detection mode). The same inconsistency was reported earlier in studies on the global methylation level of a unicellular eukaryote—*P. tetraurelia* [42]; for which the immunoassay readouts were 0.2–2.2% of 5 mC, but DNA mass spectrometry and bisulfite sequencing analyses revealed that levels were actually below the limit of detection. Further, detailed studies are needed to resolve those discrepancies.

## 5. Conclusions

In this study, we report on differential DNA methylation levels in the genome of *Y. lipolytica* subjected to repeated subculturing in RB cultivations. Primarily, we demonstrated that 5 mC modification can be detected using a commercial immuno-assay and that the modifications are present in *Y. lipolytica*’s genome (at ~0.5% 5 mC level). On the other hand, we did not observe any epigenetic response of *Y. lipolytica* to HS treatment, in terms of 5 mC frequency. Interestingly, we identified a general phenomenon of decreased 5 mC level in *Y. lipolytica*’s genome in the stationary phase of growth, when compared to a late-exponential epigenome. Such a trend has been previously observed for *Y. lipolytica*’s genome assayed through GC-MS [15]. While this study provides an insight into the subculturing stress response and adaptation to the stress at the epigenetic level by *Y. lipolytica*, it also leaves an open question of the inability to detect any genomic DNA methylation level above the negative control (either in CpG context or context-less) through ONT whole-genome sequencing. The conducted global genome sequencing suggests that 5 mC modification is either rare or non-existent in the *Y. lipolytica* genome, which is contradicted by the immunoassay, normalized with the same negative control.

## Data Availability

The data presented in this study are available in the article and Supplementary Materials. ONT sequencing data were deposited to SRA under PRJEB44499 accession.

## Supplementary Materials

The following are available online at https://www.mdpi.com/article/10.3390/microorganisms9091798/s1. Supplementary File 1: Figure S1. Kinetics of glycerol utilization and production of the main metabolites by *Y. lipolytica* W29 strain in batch cultivations (single batch period). Curves indicate the mean values of 3 biological replicates and 3 technical replicates are shown. SD < 10%. Supplementary File 2: Figure S2. Kinetics of glycerol utilization and production of the main metabolites by *Y. lipolytica* W29 strain in repeated batch cultivations (single batch period of 26 h; 3 subsequent batches are shown). Curves indicate the mean values of 3 biological replicates and 3 technical replicates are shown. SD < 10%. Supplementary File 3: Table S1. Details of statistical analysis.
