# Supplementary material for: Epigenetic Response of Yarrowia lipolytica to Stress: Tracking Methylation Level and Search for Methylation Patterns via Whole-Genome Sequencing
Source: Microorganisms. 2021 Aug 24;9(9):1798. doi: 10.3390/microorganisms9091798 (PMC8471669; doi:10.3390/microorganisms9091798)
Supplement: Supplementary file 1 [file microorganisms-09-01798-s001.zip › Supplementary File 3.pdf]

S1 Table

S1A Table Details of ANOVA statistical analysis for data collected from repeated batch cultivations. Methylation level in % 5mC was the input variable.

Effect	One-dimensional results for each variable. Sigma-restricted parameterization. Decomposition of effective hypotheses				
	Degrees of Freedom	Methylation			
		SS	MS	F	p
**Constant Term**	1	307,1237	307,1237	435,4845	0
**HS *vs* control**	1	0,4376	0,4376	0,6204	0,434758
**Cycle number**	**2**	**11,6161**	**5,8081**	**8,2355**	**0,000841**
Time after HS	2	2,614	1,307	1,8533	0,167761
**Error**	48	33,8518	0,7052		
**Total**	53	48,5195			

S1B Table Details of Tukey multiple comparison test for data collected from repeated batch cultivations. Methylation level in % 5mC was the input variable.

No	Tukey HSD Test; Variable: Methylation; Approximate probabilities for post hoc tests				
	**Cycle Number**	{1}	{2}	{3}	
1	1		0,005678	0,001603	
2	2	0,005678		0,895592	
3	3	0,001603	0,895592		
No	Tukey HSD Test; Variable: Methylation; Approximate probabilities for post hoc tests				
	**Time after HS**	{1}	{2}	{3}	
1	0		0,255197	0,205908	
2	A	0,255197		0,991399	
3	B	0,205908	0,991399		
No	Tukey HSD Test; Variable: Methylation; Approximate probabilities for post hoc tests				
	**HS *vs* C**	{1}	{2}		
1	C		0,434900		
2	HS	0,434900			
