# Supplementary figures and images for: Epigenetic Response of Yarrowia lipolytica to Stress: Tracking Methylation Level and Search for Methylation Patterns via Whole-Genome Sequencing
Source: Microorganisms. 2021 Aug 24;9(9):1798. doi: 10.3390/microorganisms9091798 (PMC8471669; doi:10.3390/microorganisms9091798)

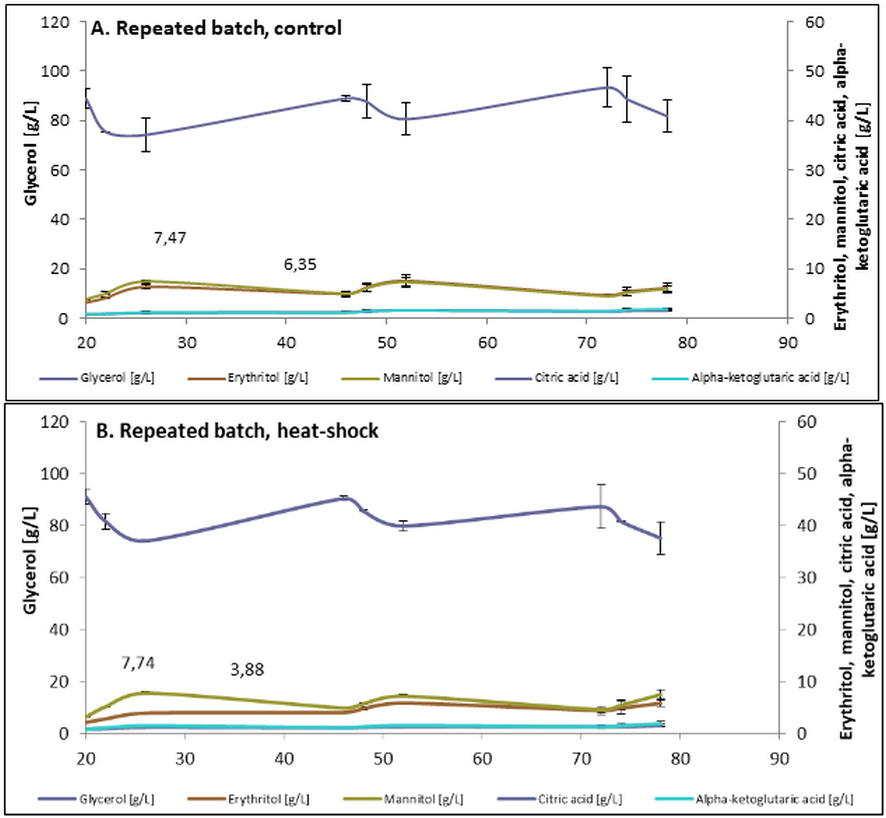

Supplement: Supplementary file 1 [file microorganisms-09-01798-s001.zip › Supplementary File_2.tif]

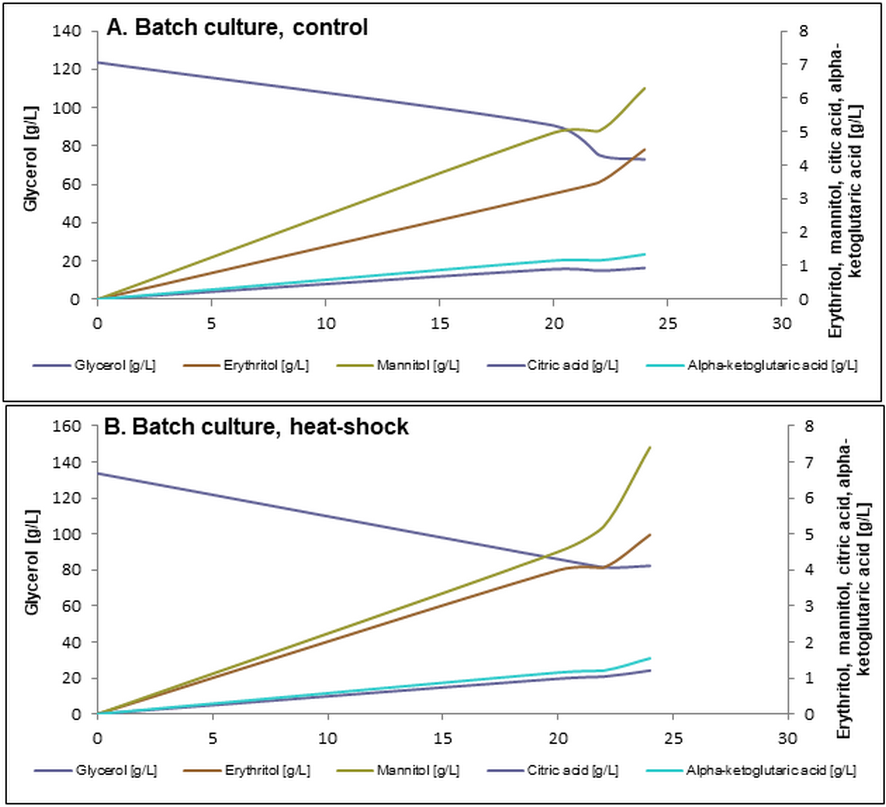

Supplement: Supplementary file 1 [file microorganisms-09-01798-s001.zip › Supplementary File 1.tif]
